# Photoreceptor Guanylate Cyclase (*GUCY2D*) Mutations Cause Retinal Dystrophies by Severe Malfunction of Ca^2+^-Dependent Cyclic GMP Synthesis

**DOI:** 10.3389/fnmol.2018.00348

**Published:** 2018-09-25

**Authors:** Hanna Wimberg, Dorit Lev, Keren Yosovich, Prasanthi Namburi, Eyal Banin, Dror Sharon, Karl-Wilhelm Koch

**Affiliations:** ^1^Department of Neuroscience, Biochemistry Group, University of Oldenburg, Oldenburg, Germany; ^2^The Rina Mor Institute of Medical Genetics, Wolfson Medical Center, Holon, Israel; ^3^Sackler School of Medicine, Tel Aviv University, Tel Aviv, Israel; ^4^Department of Ophthalmology, Hadassah-Hebrew University Medical Center, Jerusalem, Israel

**Keywords:** GUCY2D mutation, Leber congenital amaurosis, cone-rod dystrophy, guanylate cyclase, RD3 protein, GCAP

## Abstract

Over 100 mutations in *GUCY2D* that encodes the photoreceptor guanylate cyclase GC-E are known to cause two major diseases: autosomal recessive Leber congenital amaurosis (arLCA) or autosomal dominant cone-rod dystrophy (adCRD) with a poorly understood mechanism at the molecular level in most cases. Only few mutations were further characterized for their enzymatic and molecular properties. GC-E activity is under control of neuronal Ca^2+^-sensor proteins, which is often a possible route to dysfunction. We investigated five recently-identified GC-E mutants that have been reported in patients suffering from arLCA (one large family) and adCRD/maculopathy (four families). Microsatellite analysis revealed that one of the mutations, c.2538G > C (p.K846N), occurred *de novo*. To better understand the mechanism by which mutations that are located in different GC-E domains develop different phenotypes, we investigated the molecular consequences of these mutations by expressing wildtype and mutant GC-E variants in HEK293 cells. Analyzing their general enzymatic behavior, their regulation by Ca^2+^ sensor proteins and retinal degeneration protein 3 (RD3) dimerization domain mutants (p.E841K and p.K846N) showed a shift in Ca^2+^-sensitive regulation by guanylate cyclase-activating proteins (GCAPs). Mutations in the cyclase catalytic domain led to a loss of enzyme function in the mutant p.P873R, but not in p.V902L. Instead, the p.V902L mutation increased the guanylate cyclase activity more than 20-fold showing a high GCAP independent activity and leading to a constitutively active mutant. This is the first mutation to be described affecting the GC-E catalytic core in a complete opposite way.

## Introduction

Signal transduction in vertebrate rod and cone photoreceptor cells is characterized by an interplay between the two second messengers Ca^2+^ and cGMP. Cyclic nucleotide-gated (CNG)-channels in the cell membranes of rod and cones are kept open by cGMP and close, when cGMP is hydrolyzed upon illumination leading to the hyperpolarization of the cell. A second consequence of illumination is the decrease of the cytoplasmic Ca^2+^ level providing negative feedback regulation (Arshavsky and Burns, 2012; Koch and Dell’Orco, 2015). The photoreceptor guanylate cyclase GC-E (alternatively dubbed retGC1 or ROS-GC1) represents a key enzyme in phototransduction, important for the restoration of cytoplasmic cGMP and return to the dark state of the cell. Synthesis of cGMP by GC-E is regulated by guanylate cyclase-activating proteins (GCAPs), which are activated by decreasing Ca^2+^ concentrations in the cell (Palczewski et al., 2004; Dizhoor et al., 2010; Koch and Dell’Orco, 2013).

Mutations in the *GUCY2D* gene coding for GC-E lead to severe retinal diseases in humans and mainly autosomal dominant cone-rod dystrophy (adCRD) or autosomal recessive Leber congenital amaurosis type 1 (arLCA1; Duda and Koch, 2002). For adCRD, *GUCY2D* mutations are the major cause (Sharon et al., 2018). In CRD, degeneration starts in the cones and leads to loss of the central visual field due to the high presence of cones in the macula of a non-affected retina. CRD can lead to complete blindness, when degeneration of rods follows those of cones (Hamel, 2007; Berger et al., 2010). The LCA1 phenotype appears even more severe, with photoreceptor function loss and blindness emerging very early in life (den Hollander et al., 2008; Boye, 2014a,b). Another gene that is involved in the pathogenesis of LCA (type 12) is *rd3* coding for the retinal degeneration 3 (RD3) protein, which is an effective inhibitor of GCAP-mediated activation of GC-E and is involved in trafficking of GC-E from the inner to the outer segment in photoreceptors (Lavorgna et al., 2003; Friedman et al., 2006; Azadi et al., 2010; Peshenko et al., 2011).

While more than a hundred mutations in the *GUCY2D* gene were described, a link to functional consequences in the enzyme was set just for a small number, compared to the large number of known mutations. Most previous functional studies focused on mutations in the dimerization domain (DD) of the GC-E, which harbors a so-called “mutation hot spot region” (Wilkie et al., 2000; Kitiratschky et al., 2008; Zägel et al., 2013; Dizhoor et al., 2016).

In this work, we attempt to biochemically characterize some recently identified mutations and relate the phenotype to functional impairments of the enzyme. While two mutations are positioned in the DD in close vicinity to the hot spot region (p.E841K and pK846N; Lazar et al., 2014), three other mutations are located in other GC-E domains. For example, the mutation p.A710V leading to arLCA (Gradstein et al., 2016) is located in the kinase homology domain of the enzyme and two further mutations in the catalytic domain of GC-E (p.P873R) cause either adCRD or are found in a heterozygous state in an isolated case with CRD (p.V902L; both are not published so far).

Our functional analysis using recombinant proteins in heterologous expression systems showed different effects on enzyme activity due to localization in the various regions of the GC-E. Mutations in the DD are known to cause CRD and often lead to a change in Ca^2+^-sensitive regulation of the protein, which we also observed for the mutants E841K and K846N. Thus, both GC-E mutant forms needed higher Ca^2+^ concentrations to shut off enzyme activity. In contrast, the A710V and P873R mutations showed no enzyme activity at all (basal or GCAP-activated). However, a strong increase in enzyme activity was found for the V902L mutant by directly affecting the catalytic mechanism of the enzyme. This was rather unexpected, because other described mutations in the GC-E catalytic domain drastically decrease GC-E activity causing a LCA1 phenotype.

These results provide a route for better understanding the negative effects of *GUCY2D* mutations in photoreceptor cell physiology. Differences in biochemical key properties of GC-E mutants might help us to understand why some GC-E mutations lead to a LCA phenotype while others result in CRD.

## Materials and Methods

### Clinical Analysis, Mutation Detection, Cloning of GC-E Mutants With Site-Directed Mutagenesis

The study protocols adhered to the tenets of the Declaration of Helsinki and received approval from the local Ethics Committee of Hadassah Medical Center. Prior to donation of a blood sample, a written informed consent was obtained from all individuals who participated in this study, after explanation of the nature and possible consequences of the study. Ocular evaluation included a comprehensive ophthalmologic exam, Goldmann perimetry, electroretinography (ffERG), electro-oculography (EOG), color vision testing, color and infrared fundus photos, optical coherence tomography (OCT), and fundus autofluorescence (FAF) imaging were performed.

Sanger sequencing of PCR products was used to screen all exons of *GUCY2D* for mutations. The primers are listed in **Supplementary Table S1**. In addition, we used four sets of microsatellite markers flanking the GUCY2D gene (**Supplementary Table S1**). For each set, the forward primer was labeled with a FAM fluorescence dye.

To create the five desired GC-E mutants, the wildtype (WT) sequence was cloned into a pIRES2-eGFP vector and used as a template (Zägel et al., 2013). The Q5^®^ Site-Directed Mutagenesis Kit (New England Biolabs, Ipswich, MA, United States) was used to introduce point mutations in the GC-E sequence. Instructions according to the manufacturer’s protocol were followed. The primers used to produce the mutants are listed in **Supplementary Table S1**.

The obtained clones were verified by full-length sequencing of the GC-E coding region.

### Stable Transfection and Expression of GC-E Mutants in HEK-293T Cells

HEK 293T cells were used for the expression of GC-E WT protein and the five mutants. For each clone, a stable cell line was created. Cells were transfected with PolyFect (Qiagen, Hilden, Germany) and stable clones were selected via G418 antibiotic resistance. Positive clones were recognized by GFP fluorescence and were validated by western blotting with a GC-E specific antibody (following the protocol as described recently; Zägel et al., 2013). Confluent HEK cells were harvested. The cells of one 10 cm plate were transferred into a 15 ml tube and centrifuged for 5 min at 1000 ×*g*. Cell pellets were washed with PBS, transferred into a 1.5 ml tube, and centrifuged again for 5 min at 12,000 ×*g*. The pellets were frozen at -80°C until further use. Determination of protein concentration in the presence of lipids was performed according to a standard Amido Black assay (Kaplan and Pedersen, 1985).

### Expression and Purification of GCAP1, GCAP2, and RD3 in *E. coli*

Bovine myristoylated GCAP1 and GCAP2 were expressed in *Escherichia coli* and purified via size-exclusion and anion-exchange chromatography. The detailed procedure was described earlier (Hwang et al., 2003; Koch and Helten, 2008). Human *rd3* was cloned into a petM11 vector, creating a His_6_-tagged construct. RD3 was expressed and purified from *E. coli*. Ni-affinity chromatography was used for purification. The protein was stored in 10% glycerol at –80°C. The detailed purification protocol was described recently (Wimberg et al., 2018).

### GC-E Activity Assays

To analyze the effect of point mutations on GC-E function, the enzymatic activity of GC-E mutants was measured in comparison to the WT. HEK cell pellets were resuspended in 500 μl of 10 mM Hepes/KOH pH 7.4, 1 mM DTT, and protease inhibitor cocktail. The suspension was incubated for 30 min on ice. Cell lysis was performed using a syringe with a 0.6 mm tip. After centrifugation (5 min, 13,000 × *g*), the cell pellet was resuspended in 100 μl of 50 mM Hepes/KOH pH 7.4, 50 mM KCl, 20 mM NaCl, and 1 mM DTT. For each sample, 10 μl of these membrane suspensions were used. They were mixed with 20 μl of a GCAP1 or GCAP2 solution (5 μM) that was previously adjusted to different free Ca^2+^ concentrations using a Ca^2+^/EGTA buffer system exactly as described before (Hwang et al., 2003; Koch and Helten, 2008; Zägel et al., 2013). Samples were pre-incubated for 5 min at room temperature. Reaction was started by adding 20 μl of 2.5× GC-buffer (75 mM Mops/KOH pH 7.2, 150 mM KCl, 10 mM NaCl, 2.5 mM DTT, 8.75 mM MgCl_2_, 2.5 mM GTP, 0.75 mM, and 0.4 mM Zaprinast). The reaction mixtures were incubated for 5 min at 30°C. Reaction was stopped by adding 50 μl 0.1 M EDTA and 5 min of incubation at 95°C. Samples were centrifuged for 10 min at 13,000 ×*g*. Supernatants were analyzed for the amount of produced cGMP by RP-HPLC using a LiChrospher^®^ 100 RP-18 (5 μm) column (Merck, Darmstadt, Germany) exactly as described (Hwang et al., 2003; Koch and Helten, 2008; Zägel et al., 2013). Inhibition of GCAP-mediated activation of GC-E variants by RD3 was tested by adding increasing RD3 concentrations (0–500 nM) to the reaction mixture. Further, we tested whether the V902L mutant shows any GCAP-dependent change in activity by varying GCAP concentrations in the range from 0.25 to 10 μM (free Ca^2+^ buffered to 1.7 nM).

### GC-E Localization in HEK-293T Cells

HEK cells were grown on coverslips in a 24-well plate. Transfection was performed using polyethylenimine (PEI) at 80% cell confluence; 0.5 μg DNA were mixed with 2 μg PEI in DMEM without supplements and incubated for 10 min at 20°C. Subsequently, samples were added to the cells and incubated for 48 h at 37°C, 5% CO_2_. Cells were washed with PBS, fixed with 4% paraformaldehyde (PFA) in PBS for 10 min and again washed three times with PBS. Cells were incubated with 5% NGS (normal goat serum) in PBS pH 7.4 with 0.1% Triton X-100 for 1 h at room temperature. Primary antibodies were added and incubated for 24 h at 4°C. GC-E was detected by an anti-GC-E antibody (1:100, rabbit polyclonal H-225 named anti-ROS-GC1, Santa Cruz Biotechnology, Dallas, TX, United States). For staining of the endoplasmic reticulum (ER), an anti-Na^+^/K^+^-ATPase (1:200, mouse monoclonal H-3, Santa Cruz Biotechnology, Dallas, TX, United States) antibody was used. Cells were washed three times with PBS and incubated with secondary antibodies in PBS pH 7.4 with 0.1% Triton-X100 for 2 h at room temperature [donkey anti rabbit conjugated to Fura350, 1:200, Thermo Fisher Scientific (Invitrogen), Waltham, MA, United States; goat anti-mouse conjugated to Dylight594, 1:500, Thermo Fisher Scientific, Waltham, MA, United States]. Again, cells were washed with PBS and sealed with Fluoromount-G (Southern Biotech, Birmingham, AL, United States). The staining was analyzed using a Zeiss Axiophot fluorescence microscope.

## Results

### Genetic Screening and Clinical Assessment

**FIGURE 1:**
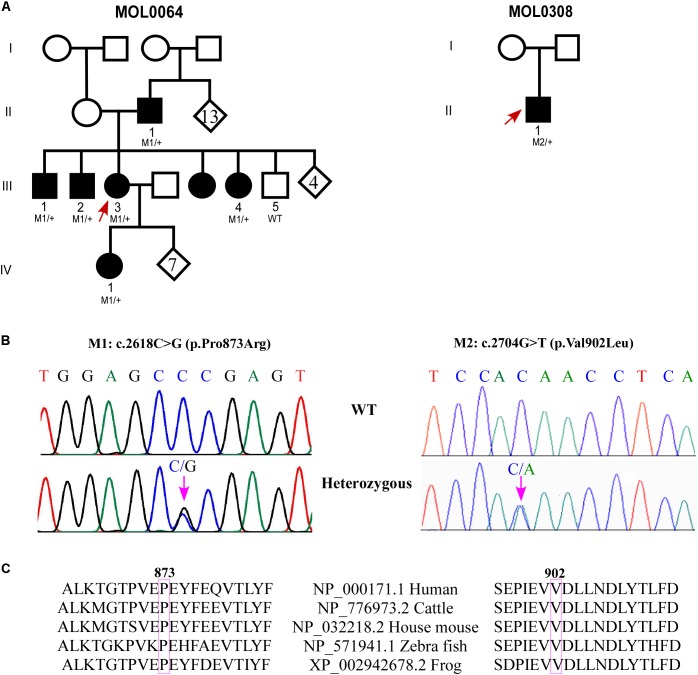
Genetic analysis of families with GUCY2D variants. **(A)** Pedigrees of MOL0064 and MOL0380. Affected individuals are marked with a filled symbol. Index cases are marked by arrows. When available, the genotype is depicted below the individual symbol. **(B)** Chromatograms of novel missense variants in *GUCY2D*. **(C)** Sequence alignment of GUCY2D proteins in the variants regions.

Family MOL0064 includes seven individuals affected with adCRD (**Figure 1A**), four of whom participated in the study and suffered from early-onset retinal degeneration (**Table 1**). ERG performed in four affected individuals showed extinguished or severely reduced cone responses (**Table 1**) and extinguished rod responses. All patients suffered from nystagmus and low visual acuity. Sanger sequencing of genes involved in inherited retinal diseases including the *GUCY2D* region encoding GC-E DD, revealed a novel heterozygous missense variant (c.2618C > G, p.Pro873Arg; **Figure 1B**) in the four affected individuals who participated in the study. This variant is absent from databases (gnomAD, ExAC), is predicted to be pathogenic according to a variety of prediction tools (MutationTaster^1^; PolyPhen-2^2^; and SIFT^3^), and is highly conserved (**Figure 1C**). The mutation was validated in our clinical lab and it was not found in the healthy brother.

**Table 1:** Clinical data of patients with *GUCY2D* mutation.

Pt. Number	Age (years)	Best Corrected Visual Acuity (age)*	Full Field ERG Results			EOG (%)	Comments
			Cone Flicker - 30Hz, IT in msec	Mixed Cone-Rod Response (μV)	Rod Response - Blue Light (b, μV)		
MOL0064 II:1	53		Extinguished	Extinguished	Extinguished		Nystagmus
MOL0064 III:2	22		Extinguished	Extinguished	Extinguished		Nystagmus; abnormal color vision
MOL0064 III:3	31	0.33	7 (42)	55	Extinguished	125	Nystagmus
	34	0.25					
MOL0064 IV:1	8	0.1	Extinguished	Extinguished	Extinguished		
MOL0308 II:1	1	0.07	Extinguished	Very low	Extinguished		Congenital nystagmus, photophobia Scotopic lines
	4	0.1	Extinguished	Very low			
	7		Extinguished			
	11			Extinguished	
MOL0430-1	24^#^ 29		34 (39.3)	a=230, b=229	219	232	Tritamopia
			21 (38.5)	a=174, b=241	176	152	
MOL0508-1	25		46 (32.9)	a=217, b=389	298	191	Severe Tritanopia, maculopathy


*NA, not available; IT, implicit time. ^∗^Age at ERG testing is indicated. If measurements were performed at an age that is different from the ERG testing age, the age is indicated in parentheses. Best corrected visual acuity is presented in decimal values as an average of the two eyes. ^#^Published previously.*

MOL0308 (**Figure 1A**) includes an affected child with early-onset CRD. Following negative analysis for the known CRD mutations in the relevant population, we performed whole exome sequencing on his DNA sample. The analysis revealed a novel heterozygous missense variant in *GUCY2D*: c.2704G > T; p.V902L (**Figure 1B**). This variant is absent from databases (gnomAD, ExAC), is predicted to be pathogenic according to a variety of prediction tools, and is highly conserved (**Figure 1C**). The mutation was validated in our clinical lab and it was not found in the healthy mother. Unfortunately, the father’s DNA sample was not available.

MOL0508 includes two affected individuals, an index case and her mother, with macular degeneration and cone dystrophy. We have previously reported (Lazar et al., 2014) that we identified a heterozygous variant (c.2521G > A, p.E481K) in *GUCY2D*.

MOL0430 includes an affected male with CRD who was found to be heterozygous for the c.2538G > C (p.K846N) variant in *GUCY2D* as we previously reported (Lazar et al., 2014). For the current study, we were able to recruit additional family members, including both parents and three siblings, all are unaffected and none carries the variant in *GUCY2D*. Haplotype analysis using four microsatellite markers flanking *GUCY2D* revealed that the index case (individual II:1 in **Supplementary Figure S1**) was the only sibling to inherit the paternal haplotype 141-205-251-272 while his three siblings inherited the counter allele. However, the index case shares the maternal haplotype 161-199-255-277 with two of his unaffected siblings (II:3 and II:4), indicating a paternal *de novo* mutation.

### Cloning and Stable Expression of GC-E Mutants in HEK-293T Cells

**FIGURE 2:**
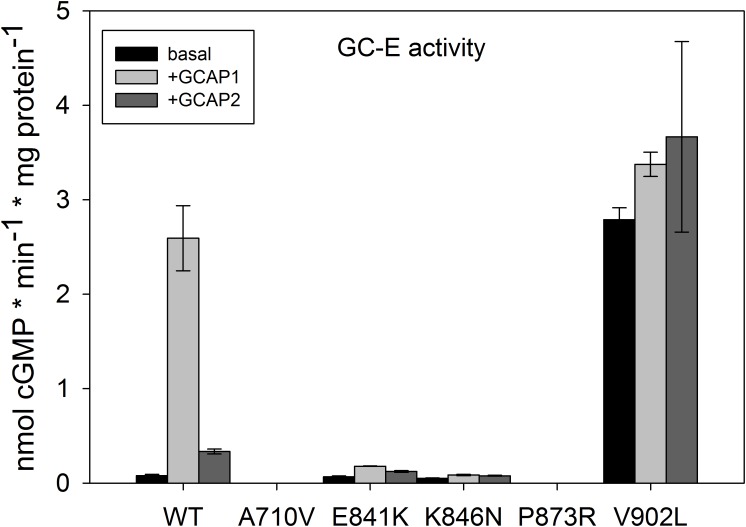
Basal and GCAP-mediated activity of GC-E wildtype and mutants. Maximal activity of GC-E wildtype and mutants was measured via a GC-activity assay. The amount of produced cGMP per minute and μg protein was calculated and compared between the wildtype and mutants. GC-E activation by GCAPs (5 μM) was detected at low [Ca^2+^] (1.7 nM). Each data point represents the mean value of three replicates with the standard deviation. The experiment was repeated twice with similar results.

Point mutants of GC-E WT were successfully created by site-directed mutagenesis as proven by full-length sequencing. Immunohistochemistry and immunoblot analyses confirmed expression of WT and mutant GC-E in stable cell lines as shown in **Supplementary Figure S2**. Protein expression levels of GC-E variants were similar, when the same amounts of total protein (10 μg of cell homogenates) were loaded on a gel. Samples shown in **Supplementary Figure S2** were used for the experiment displayed in **Figure 2**, which compares activity levels of WT and mutant GC-E forms.

Cellular localization in HEK cells was the same for GC-E mutants and GC-E WT and was visible in the ER in agreement with previous observations (Peshenko et al., 2008; Zägel and Koch, 2014). Cellular localization was analyzed by immunostaining using an anti-Na^+^/K^+^-ATPase antibody as ER marker (red, **Supplementary Figure S3B**). Localization of GC-E was detected with a specific antibody (blue, **Supplementary Figure S3C**). GFP signal (green, **Supplementary Figure S3A**) indicated successfully transfected cells. Localization of GC-E in the ER became visible in the overlay (magenta, **Supplementary Figure S3D**).

### Activity of GC-E Wildtype and Mutants

In order to gain insight into the structure-function relationship of retinal disease-causing mutations in GC-E, we investigated three critical parameters of the enzymatic activity profile of GC-E: (1) increase of GC-E activity in the presence of Ca^2+^-free/Mg^2+^-bound GCAP1 and GCAP2; (2) inhibitory effect of RD3, when GC-E is in the activated state in complex with GCAP1 or GCAP2; and (3) Ca^2+^-sensitive activation profile of GC-E.

All mutants showed a severe impairment of normal GC-E activity (**Figure 2**), but type and impact of the disturbance differed among all mutants. GC-E A710V and P873R had no measurable activity. The expression of A710V in these samples was much lower than that of WT GC-E and the other mutants. To exclude that the lack of measurable activity of A710V is due to low expression levels, we created a new stable cell line for A710V that showed higher expression levels (**Supplementary Figure S2B**). But even with the highest expression level of A710V, we did not detect any activity of this mutant. The two mutations in the DD near the hot spot region exhibited drastically decreased activity in the presence and absence of GCAP1 and GCAP2, but still were able to switch from a basal enzymatic state to the GCAP-mediated activation state. Most surprisingly, the V902L mutation resulted in high basal activity that did not increase in the presence of GCAP2 and increased only to a small extent in the presence of GCAP1 (**Figure 2**). Thus, the V to L exchange in position 902 in the cyclase catalytic domain transformed the enzyme into a constitutively active conformation.

### Inhibitory Effect of RD3

**FIGURE 3:**
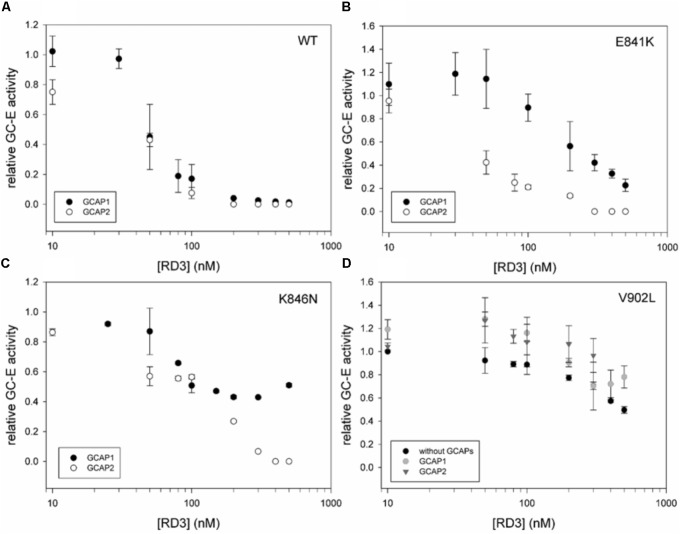
Inhibition of GCAP activated GC-E wildtype and mutants by RD3. **(A)** GC-E wildtype. **(B)** GC-E mutant E841K. **(C)** GC-E mutant K846N. **(D)** GC-E mutant V902L. GC-E from HEK cells was incubated with either GCAP1 or GCAP2 (5 μM) or no GCAPs (V902L) and increasing concentrations of RD3 (0–500 nM) at low [Ca^2+^] (1.7 nM). The GC-E activity at a concentration of 0 nM RD3 was set to 1 and the relative activity was calculated due to the amount of produced cGMP. Each data point represents the mean value of three replicates with the standard deviation. The experiment was repeated with similar results.

Retinal degeneration protein 3 is a strong inhibitor of GCAP-mediated activation of GC-E (Peshenko et al., 2011) showing half-maximal inhibition in the lower nanomolar range reaching complete inhibition > 100 nM (**Figure 3A**). The inhibitory profiles of RD3 inhibition were nearly identical for GCAP1 and GCAP2 mediated activation of GC-E. In comparison to WT GC-E, we tested the three mutants that have residual (E841K and K846N) or constitutive activity (V902L) by setting up the same titration series with purified RD3 (**Figures 3B–D**). Inhibition by RD3 differed in all mutant cases from inhibition of the WT, except for E841K in the presence of GCAP2. When E841K was tested with GCAP1 and increasing concentrations of RD3, half-maximal inhibition is shifted to higher concentrations of RD3 (**Figure 3B**). A shift in half-maximal inhibition was also observed for the mutant K846N (**Figure 3C**), but the GC-E activity was only suppressed to 50% at around 500 nM RD3 in the presence of GCAP1 and to more than 90% in the presence of GCAP2.

Most interestingly, the constitutively active mutant V902L stayed active even in the presence of high concentrations of RD3, which are sufficient for completely suppressing GCAP-mediated activity of the WT (**Figures 3A,D**). The presence or absence of GCAPs did not lead to a significant difference in the inhibitory profiles.

### Ca^2+^-Sensitive Activation of GC-E Mutants

**FIGURE 4:**
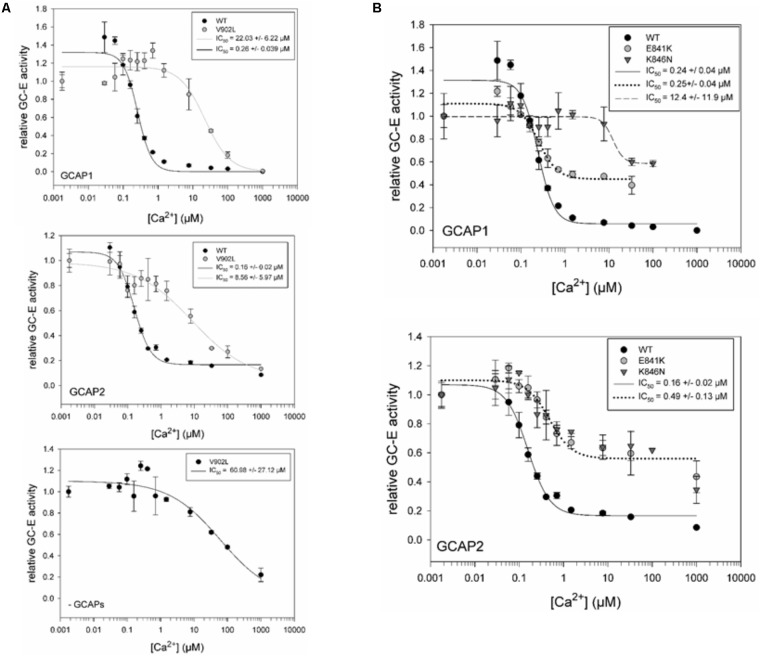
IC_50_ measurements for GC-E wildtype and mutants. **(A)** Comparison of GC-E wildtype and mutant V902L, activated by GCAP1 (upper panel) and GCAP2 (middle panel). An additional Ca^2+^ titration of the mutant V902L (lower panel) without GCAPs was performed. **(B)** Comparison of GC-E wildtype with the mutants E841K and K846N. GC-E from HEK cells and GCAPs (5 μM) were incubated at different [Ca^2+^] ranging from 1.7 nM to 1 mM. The relative GC-E activity was calculated via the amount of produced cGMP. The GC-E activity at the lowest [Ca^2+^] was set to 1. IC_50_ values are indicated in the graph and represent the half-maximal inhibition by Ca^2+^. Each data point represents the mean value of three replicates with the standard deviation. The experiment was repeated for GC-E wildtype and V902L with similar results. The data were fitted in SigmaPlot 11.0 with a Hill 3 parameter fit. The inset shows IC_50_ values with the standard error of the fitting.

The constitutive activation of the V902L mutant seems to mimic the activation of GCAPs. GCAP1, but not GCAP2, caused a slight increase on top of the activity without GCAPs (see above and **Figure 2**). Since the experiment in **Figure 3** did not show any possible distortion of the Ca^2+^-sensitive regulation, we tested for an effect in the presence of GCAP1, GCAP2, and without GCAPs present. The activation profile of the V902L mutant shifted about 100-fold from an IC_50_ of 0.26 (WT) to an IC_50_ of 22.03 μM free Ca^2+^ (**Figure 4A**, upper panel), which further adds to the severe dysregulation of this GC-E mutant. An IC_50_ shift for GCAP2 was also observed, but to a lesser extent. Here the value shifted from 0.16 to 8.56 μM free Ca^2+^ (**Figure 4A**, middle panel). Interestingly, when no GCAPs are present, the V902L mutant was inhibited by increasing free Ca^2+^ resulting in an IC_50_ of about 61 μM free Ca^2+^ (**Figure 4A**, lower panel). Apparently, this inhibitory effect was not mediated by GCAPs. Instead, it could originate from a competition of Ca^2+^ with Mg^2+^ that is a necessary co-factor of GTP in the catalytic site. However, since Mg^2+^ concentrations in the assay medium are relatively high with 1 mM free Mg^2+^, this effect is normally visible at millimolar Ca^2+^ concentrations.

The mutants E841K and K846N shared some characteristic features in their Ca^2+^-sensitive regulation in the presence of GCAPs (**Figure 4B**). They showed no complete inhibition at high free Ca^2+^ concentrations of 50–100 μM, a shift in the IC_50_ for K846N with GCAP1 and for E841K and K846N with GCAP2. No shift was observed for E841K in the presence of GCAP1. **Table 2** summarizes data on biochemical properties of the GC-E mutants.

**Table 2:** mutations characterized in this study.

Mutation name	References	Inheritance pattern	Family #	Phenotype	GC-E activity	IC_50_ GCAP1 GCAP2	Inhibition by RD3	Localization (HEK cell model)
p.A710V	Gradstein et al., 2016	AR		LCA	No activity	–	–	ER
c.2521G > A (p.E841K)	Lazar et al., 2014	AD	MOL0508 (two patients)	Maculopathy	Decreased	0.25 ± 0.04 μM 0.49 ± 0.13 μM activity left > 1 mM Ca^2+^	Less effective	ER
c.2538G > C (p.K846N)	Lazar et al., 2014	Isolate (*de novo*)	MOL0430 (one patient)	CRD	Decreased	12.4 ± 11.9 μM >2.0 μM^∗^ Activity left > 1 mM Ca^2+^	Less effective	ER
c.2618C > G (p.P873R)	Novel	AD	MOL0064 (seven patients)	CRD	No activity	–	–	ER
c.2704G > T (p.V902L)	Novel	Isolate	MOL0308	CRD	Increased (basal) Normal (GCAP1) Increased (GCAP2)	22.03 ± 6.22 μM 8.56 ± 5.97 μM 60.98 ± 27.12 μM (w/o GCAPs)	Less effective	ER


*^∗^Data could not be fitted to a Hill 3 parameter function. IC_*50*_ value represents an approximation derived from visual inspection of the graphics.*

### Effect of Increasing GCAP Concentration on the V902L Mutant

**FIGURE 5:**
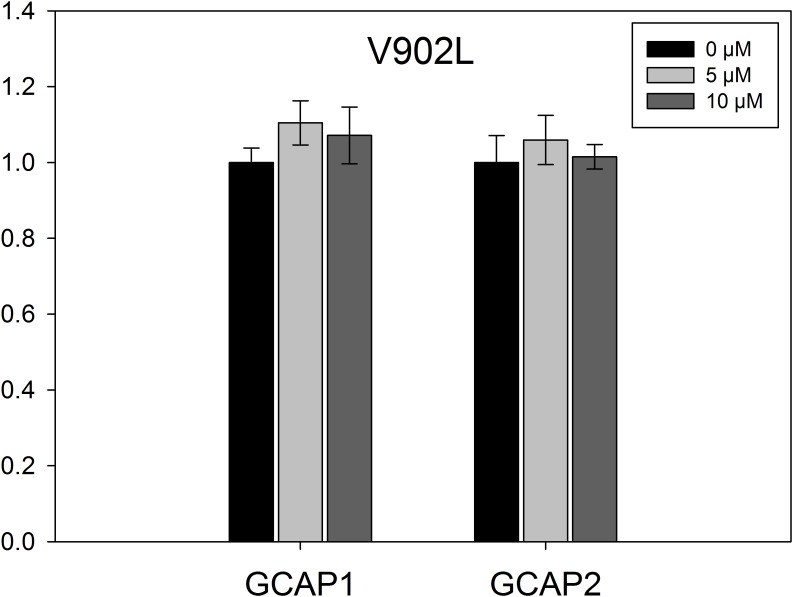
EC_50_ measurements for mutant V902L. The GC-E mutant V902L was incubated with increasing concentrations of GCAP1 or GCAP2 ranging from 0 to 10 μM. GC-E activity was calculated via the amount of produced cGMP measured by HPLC. The GC-E activity at 0 μM GCAP was set to 1. Each data point represents the mean value of three replicates with the standard deviation. The experiment was repeated with similar results.

Experiments in **Figure 2** indicated a GCAP independent high activity for the V902L mutant. We assayed the activity of the V902L mutant with increasing GCAP1 and GCAP2 concentrations (0–10 μM; **Figure 5**). No effect of GCAP1 or GCAP2 on the GC-E V902L mutant activity was observed, not even at high GCAP concentrations of 10 μM that are saturating concentrations for WT GC-E. Probably GCAPs have no stimulating effect on the V902L mutant, but still exhibit an inhibitory effect, as seen in the IC_50_ measurements.

## Discussion

**FIGURE 6:**
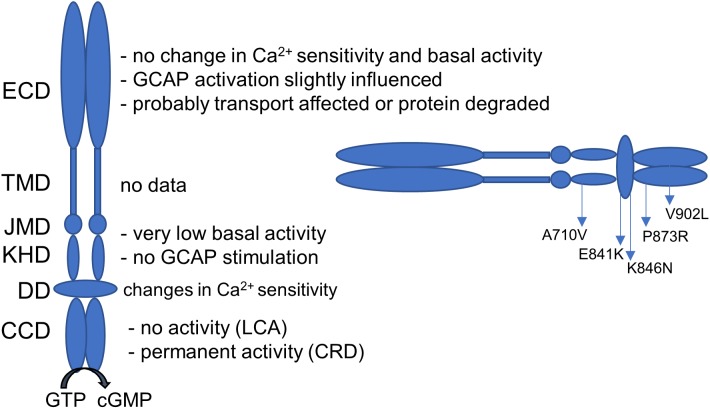
Guanylate cyclase domains and the effect on *GUCY2D* mutations on the enzyme function. Dependent on the localization, mutations can have several consequences on GC-E function. Localization of the investigated mutations is given. ECD, extracellular domain; TMD, transmembrane domain; JMD, juxtamembrane domain; KHD, kinase homology domain; DD, dimerization domain; CCD, cyclase catalytic domain.

Guanylate cyclases are expressed in two forms in human photoreceptor cells: GC-E and GC-F (Dizhoor et al., 1994; Lowe et al., 1995). They play a central role in phototransduction and mutations in the *GUCY2D* gene coding for human GC-E can lead to severe retinal diseases in humans. Inherited retinal diseases display a very heterogeneous group of disorders and the number of causative genes heads toward 300. A total number of 144 different *GUCY2D* mutations have been described so far (see Sharon et al., 2018 for a recent update). The majority (127 mutations) result in a LCA phenotype in the affected patients. While LCA-related mutations are usually recessive and null (mainly frameshift, non-sense, and splicing mutations) and can affect all domains of the GC-E enzyme, CRD mutations are mainly dominant missense clustered in a “hot-spot region” in the DD, at positions between E837 and T849 (Wilkie et al., 2000; Kitiratschky et al., 2008; Sharon et al., 2018). To answer the question why some *GUCY2D* mutations lead to a CRD phenotype and others to LCA, it is important to understand how these mutations influence the enzyme properties. Different studies already investigated the effect of *GUCY2D* point mutations. Some general findings are summarized in **Figure 6** (Rozet et al., 2001; Tucker et al., 2004; Peshenko et al., 2010; Jacobson et al., 2013; Zägel and Koch, 2014).

In this study, we characterized five different GC-E mutants. Patients with the homozygous missense mutation p.A710V showed extinct ERG responses and nystagmus. All exhibited poor vision and nyctalopia before 1 year of age. Position A710 is located in the KHD and highly conserved between species. Molecular modeling approaches implied that the mutation probably leads to a loss of GC-E helical structure, which might affect the catalytic center (Gradstein et al., 2016). Mutations affecting the JMD and KHD of the GC-E typically show no or very low basal activity and cannot be activated by GCAPs (Duda et al., 1999; Jacobson et al., 2013). These mutations may change the overall structure of the protein preventing activation of GC-E by GCAPs. Direct binding of GCAP1 could be impaired, since either its binding site or an important activity control site is located in this domain (Sulmann et al., 2017). Further, the KHD harbors a putative Mg^2+^ binding site that is part of the nucleotide (ATP) binding site and is suggested to stabilize the active conformation of the catalytic domain by multiple hydrogen bonds (Bereta et al., 2010). The mutation p.A710V is located within a structural motif of the GC-E, called ^708^WTAPELL^714^ motif, which is critical for the regulatory catalytic activity of GC-E and conserved in all membrane GCs (Duda et al., 2011). Our experiments showed that the A710V mutant lacks any enzymatic activity. This may explain the LCA phenotype in these patients, because LCA1 in general is related to a loss of GC-E function or proper expression. Interestingly, the complete deletion of the WTAPELL motif affects the GC-E to a lesser extent. The basal GC-activity was normal, but activation by GCAP1 and GCAP2 was reduced. Single point mutations W→A, T→A, P→A, and E→A did not affect the basal activity and activation by GCAPs yielded 25–50% compared to the WT (Duda et al., 2011). These less dramatic effects would probably not lead to a LCA phenotype assuming that corresponding mutant proteins are still transported to outer segments and not degraded. In contrast, the A710V exchange seems to influence the overall GC-E structure more dramatically and completely abolished its activity in our enzymatic assays.

The two DD mutants E841K and K846N exhibited typical hallmarks of activity changes in comparison to the WT guanylate cyclase as summarized in Sharon et al. (2018). These are mainly reduced basal activity and a drastically reduced activation by GCAP1 and GCAP2. The low, but remaining activation by GCAPs was shifted to higher Ca^2+^ concentrations keeping the GC-GCAP complex constitutively active (**Figures 2, 4B**). The consequence of permanent cGMP production under conditions of high cytoplasmic Ca^2+^ would open CNG-channels and increase Ca^2+^ influx. Accumulation of cGMP and disturbance in the Ca^2+^ homeostasis of the cell can have neurotoxic effects (Iribarne and Masai, 2017). This may explain the progressive CRD phenotype in patients. In line with the biochemical analysis, patients with the p.E841K or p.K846N mutations were diagnosed with maculopathy and CRD (Lazar et al., 2014). We demonstrated here that the c.2538G > C (p.K846N) mutation is a *de novo* paternal mutation, and therefore verifying the pathogenicity of this sequence variant. Only one previous *GUCY2D de-novo* mutation has been reported thus far (Mukherjee et al., 2014).

Usually, CCD mutations are associated with a complete loss of GC-E function and a LCA phenotype. The newly identified CCD mutations p.P873R and p.V902L differ in these aspects and are the first described CRD-related mutations that are found in the catalytic domain of the GC-E. Four individuals of the MOL0064 family participated in this study, showing early onset retinal degeneration and extinguished or severely reduced ERG responses. The p.P873R mutation abolished the activity of the enzyme completely and is therefore expected to be associated with a recessive LCA phenotype. The patients, however, were heterozygous for the p.P873R variant, which co-segregated perfectly in the family and no possible disease-causing mutation has been identified on the counter allele. Earlier studies showed that CCD mutants can exhibit dominant negative effects, so that the disease is also prominent in heterozygotes with milder effects (Tucker et al., 2004). This means that in these patients also GC-E WT protein is present, but to a much lesser extent.

Furthermore, RD3 mediated trafficking can be effected in LCA-related CCD mutations due to less efficient binding of RD3 to GC-E (Zulliger et al., 2015). Interestingly, all tested mutants (E841K, K846N, and V902L) were less inhibited by RD3 than WT GC-E. This indicates a possible role for RD3 in the disease development in case of *GUCY2D* mutations. Lower GC-E inhibition in photoreceptor inner segments would lead to non-balanced cGMP production in inner segments and uncontrolled activation of cGMP target proteins (e.g., protein kinase G, CNG-channels). A disturbance of the inner to outer segment trafficking (Azadi et al., 2010) would lead to lower expression levels of GC-E in the outer segment and an imbalance of cGMP levels. Future studies need to address, which consequences develop from such distortions.

The V902L variant displayed a unique biochemical phenotype. The point mutation resulted in a GCAP independent permanently active GC-E. It turned out that CRD mutations can also appear in the catalytic domain of the GC-E and they do not always lead to a loss of function. At first glance, the amino acid exchange from valine to leucine is not dramatic. Both are non-polar aliphatic amino acids with a branched chain. With the change to leucine only the side chain is prolonged from an isopropyl to an isobutyl group. Somehow, this exchange leads to structural changes in the catalytic core of the enzyme, that turns the GC-E in a GCAP independent, permanent active form. To date, no complete structure of an active membrane bound mammalian guanylate cyclase was resolved (Potter, 2011). Therefore, good predictions on the conformational changes due to the mutation cannot been made, but one can assume that the mutation causes a stabilization of the enzymatic transition state that in the WT is achieved by the binding of GCAPs. Furthermore, the constitutive activity of the V902L mutant is similar to those seen with DD mutations and high synthesis rates of cGMP would result in the permanent opening of CNG-channels and increased Ca^2+^ levels. The effect may be even more severe, because this mutant showed a very high basal activity compared to the other mutants (E841K and K846N) investigated in this study.

We here show that mutations in *GUCY2D* result in multiple effects on guanylate cyclase function and may provide a basis to develop specific therapies for patients suffering from *GUCY2D* mutations. Currently, cGMP analogs targeting protein kinase G and CNG-channels are under investigation for the treatment of retinal diseases. Compounds with sufficient efficacy could counteract photoreceptor degeneration, while interfering with photoreceptor death pathways (Vighi et al., 2018). Abnormal cGMP levels are a common feature in retinal diseases and probably also in CRD-related *GUCY2D* mutations, due to the constitute activation of guanylate cyclase. Therefore, drug treatment approaches may be possible in CRD cases caused by *GUCY2D* mutations and could include application of cGMP analogs (Vighi et al., 2018) or CNG-channel blockers (Koch and Kaupp, 1985) to counteract high cGMP levels.

Recently, the idea for gene augmentation therapy in LCA1 cases has been suggested (Aguirre et al., 2017), in light of the lack of basal activity of *GUCY2D* mutants that cause LCA, for example, the mutation p.A710V. This makes it a suitable target for gene replacement therapy, because no interfering native protein will be present. Additionally, most LCA1 patients show apparently normal fundus and some photoreceptors showing normal structure, which is required to restore vision by gene therapy. Although reports on photoreceptor degeneration in LCA1 patients are inconsistent, a recent study with patients aged from 6 months to 37 years described some rod photoreceptors with normal architecture (Perrault et al., 1999; Jacobson et al., 2013; Boye, 2014b). Adeno associated virus-based gene therapy for LCA1 gave promising results in mice leading to structural and functional improvement for at least one year (Boye et al., 2010, 2013; Boye, 2014a). For other LCA types, for example, RPE65 mutations, already promising studies employing gene therapy were performed (MacLaren et al., 2016) and gene augmentation therapy for this gene has been recently approved by the FDA. However, gene augmentation therapy might not be effective for dominant *GUCY2D* mutations that cause CRD, since the mutant allele produces a mutant protein that affects retinal function even in the presence of a normal protein that is expressed at a similar level. Other negative side effects of introducing exogenous GC-E could arise from the complexation of the GC-E target RD3. Therefore, other approaches, and mainly those abolishing the expression of the mutant allele should be considered. Recent successful *in vivo* experiments with CRISPR-Cas9 on dominant mutations, including those causing retinal diseases, bring hope for using this technique for GUCY2D dominant mutations as well (DiCarlo et al., 2018). Therefore, depending on the type of mutation in *GUCY2D*, different therapeutic modalities should be applied.

## Author Contributions

HW designed the study, planned and carried out the experiments concerning the biochemical characterization of GC-E mutants, analyzed the data, wrote the manuscript, and prepared **Figures 2–6** and **Supplementary Figures S2, S3**. HW, DS, and K-WK discussed and structured the manuscript. DL, KY, PN, and DS did the genetic analysis of the data and generated **Figure 1** and **Supplementary Figure S1**. EB performed the clinical analysis. K-WK formulated the research question, designed the study, participated in data analysis, and wrote the final paper. All authors revised the manuscript.

## Conflict of Interest Statement

The authors declare that the research was conducted in the absence of any commercial or financial relationships that could be construed as a potential conflict of interest.

## Footnotes

1 http://www.mutationtaster.org/

2 http://genetics.bwh.harvard.edu/pph2/

3 http://sift.jcvi.org/seq_submit.php
