# Supplementary material for: Photoreceptor Guanylate Cyclase (GUCY2D) Mutations Cause Retinal Dystrophies by Severe Malfunction of Ca2+-Dependent Cyclic GMP Synthesis
Source: Front Mol Neurosci. 2018 Sep 25;11:348. doi: 10.3389/fnmol.2018.00348 (PMC6167591; doi:10.3389/fnmol.2018.00348)
Supplement: Supplementary file 1 [file Table_1.docx]

**Supplementary material**

**Photoreceptor Guanylate Cyclase (GUCY2D) mutations cause retinal dystrophies by severe malfunction of Ca^2+^-dependent cyclic GMP synthesis**

Hanna Wimberg, Dorit Lev, Keren Yosovich, Prasanthi Namburi, Eyal Banin, Dror Sharon, Karl-Wilhelm Koch

**
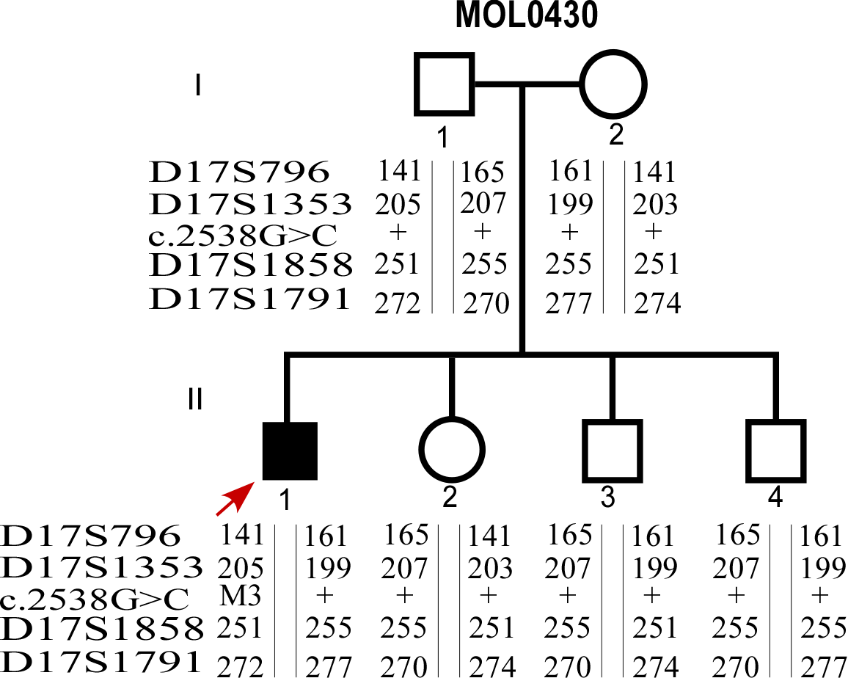
**

**Figure S1:** Haplotype analysis using four microsatellite markers for family MOL0430. Affected individual is marked with a filled symbol. Index case is marked by arrow. The haplotype of each individual is represented below the individual symbol. Numbers represent the size of the PCR product containing the microsatellite sequence, "M3" represents the mutant allele, and "+" represents the normal allele

**
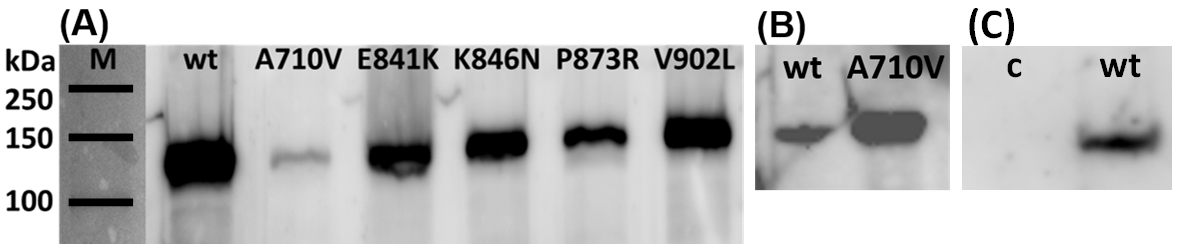
**

**Figure S2: Western blot for verification of GC-E expression in HEK cells.** The total protein amount loaded for each sample was 10 µg and was quantified by a standard Amido Black assay. GC-E was detected by a specific antibody in all samples showing a successful transfection of the cells. (A) These samples were used for the activity measurements displayed in figure 2. (B) The mutant A710V showed rather weak expression and the experiment was repeated with another preparation with sufficient protein expression shown here. (C) Negative (normal HEK cells) and positive control (GC-E wt expressing HEK cells) were tested with the GC-E antibody. M = marker, wt = wildtype, c = negative control.

**
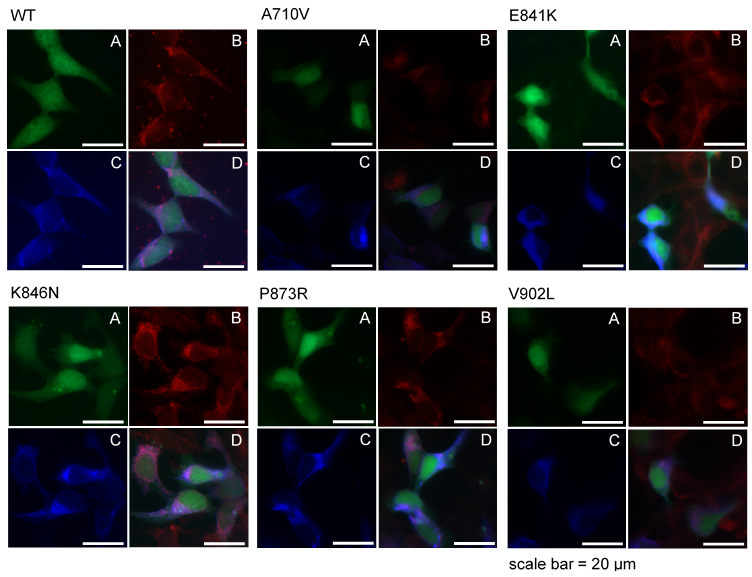
**

**Figure S3: Antibody staining and cellular localization of GC-E wildtype and mutants in HEK cells.** (A) GFP is co-expressed with the guanylate cyclase in HEK cells and indicates positive transfected cells. (B) Staining of the ER via a Na^+^/K^+^-ATPase antibody. (C) Localization of GC-E wildtype and mutants in cells via detection with a GC-E specific antibody. (D) Co-localization of the ER marker and GC-E in transfected HEK cells. Scale bar represents 20 µm.

**Table S1: Oligo sequences used in the present study.**

| **Region** | **Oligonucleotide Sequence 5'-3'** | **Product**  **Size (bp)** |
| --- | --- | --- |
| Sanger sequencing of GUCY2D exons: | | |
| Exon 2-1 | AGGCCGGGGTCTCAGTC  CTTCTTCGGCGAGCAGC | 481 |
| Exon 2-2 | GTCCCCGCTTCGAGGTAG  CATGATCACTGCTGCGGAC | 538 |
| Exon 3 | GAGGTCGGCTGGTCCTG  ATCCTCCTCTCCCTGCTTTG | 436 |
| Exon 4 | GGCTTGACAGGCAGTGAAAG  GTGGATGGTCCATGGCG | 483 |
| Exon 5-6 | AGAAGAGGCCTCCCCTGG  GGAAGGAACCAAATTTACGG | 585 |
| Exon 7 | ACCCAGGACTCTGACACCAG  CAGGGCCTGAGCATTCTTC | 217 |
| Exon 8 | CCCCATCGTGGGATTTTAAG  AAGAGAAGGCAAGGAGGGAG | 200 |
| Exon 9-10 | GATCTTGATTAACAGCCCCTTC  AAGCCCTTGAAATAATGGGG | 590 |
| Exon 11 | CTTTCTGGTGAGGGTGGGAG  TTTTCTAACTGCAGGGTGCC | 266 |
| Exon 12 | CCCTCACTGTCCCCTCATG  TGACAAGCATCTGGGATCCC | 234 |
| Exon 13-14 | GTAGATGAATGGTGGCAGCG  GATTGGGCAGGTAGGCTAGG | 680 |
| Exon 15 | ATCATTGGTTCCCACGATGT  TCTTCGCACCCATTATCTCC | 645 |
| Exon 16-17 | AATGGGTGCGAAGATCCC  CGGTGCCTCAGGACAGG | 419 |
| Exon 18-19 | CTCAGCTCACCCTTCTGACC  ACACCTGGCTTGGGTGG | 532 |
| Microsatellite markers flanking GUCY2D | | |
| D17S796F  D17S796R | CAATGGAACCAAATGTGGTC  AGTCCGATAATGCCAGGATG | 144-174 |
| D17S1353F  D17S1353R | CTGAGGCACGAGAATTGCAC  TACTATTCAGCCCGAGGTGC | 200-222 |
| D17S1858F  D17S1858R | GACATCCCCGCGTGACTC  AGGCTTGGCCTACTTTTAGCCCTTA | 247-263 |
| D17S1791F  D17S1791R | AGCTTTTGGTCAACCTG  GGGTGGGTGGAGTTAC | 270-290 |
| Primers used to produce the mutants | | |
| A710V | CTGTGGACAGtCCCGGAGCTG  CTGGTCCTCCGCTCTGGG |  |
| E841K | GCGCACGGAGaAGCTGGAGCT  TCCCGGATCAGATCCTCCAG |  |
| K846N | AGCTGGAAAAcCAGAAGACAGACCGGC  CCAGCTCCTCCGTGCGCT |  |
| P873R | CCAGTGGAGCgCGAGTACTTTG  TGTCCCCGTCTTCAAGGC |  |
| V902L | CATTGAGGTTtTGGACCTGCTC  GGCTCACTCATGGCAGAG |  |

All primer sequence is shown as 5’-3’ and for each pair of primers, the first is the forward primer and the second is reverse.
